# Supplementary material for: The experience of urgent dialysis patients with end-stage renal disease: A qualitative study
Source: PLoS One. 2022 Jan 21;17(1):e0261941. doi: 10.1371/journal.pone.0261941 (PMC8782518; doi:10.1371/journal.pone.0261941)
Supplement: S1 File — (PDF) [file pone.0261941.s001.pdf]

Characteristics of the study participants (N = 5)

	**Gender**	**Age**	**Education Level**	**Marital Status**	**Work status**	**History**	**Diagnosis**
A	male	62	secondary	Widowed	Yes	Hypertension	ESRD
B	male	60	Elementary school	Yes	Yes	Hypertension	ESRD
C	male	78	Elementary school	No	No	DM、Hypertension	ESRD
D	Female	72	illiterate	Yes	No	Hypertension、DM、Heart disease	ESRD
E	male	78	Elementary school	No	No	DM、Hypertension	ESRD
