# Supplementary material for: The experience of urgent dialysis patients with end-stage renal disease: A qualitative study
Source: PLoS One. 2022 Jan 21;17(1):e0261941. doi: 10.1371/journal.pone.0261941 (PMC8782518; doi:10.1371/journal.pone.0261941)
Supplement: Attachment — Submitted filename: PONE-D-21-04435_R1_reviewer.pdf [file pone.0261941.s004.pdf]

PLOS ONE

The experience of urgent dialysis patients with end-stage renal disease: a qualitative study

--Manuscript Draft--

**Manuscript Number:**	PONE-D-21-04435R1
**Article Type:**	Research Article
**Full Title:**	The experience of urgent dialysis patients with end-stage renal disease: a qualitative study
**Short Title:**	Mentality of patients delaying dialysis therapy
**Corresponding Author:**	KUEI-HUI CHU, PhD<br>Ching Kuo Institute of Management and Health<br>Keelung City, Taiwan TAIWAN
**Keywords:**	End-Stage Renal Disease, Hemodialysis, Qualitative Research
**Abstract:**	Purpose<br>Taiwan is among the countries with the highest global prevalence of chronic renal disease. However, when advised to undergo dialysis therapy, patients with end-stage renal disease often hesitate. Attitudes toward medication and Taiwanese cultures are the main reasons for this delay, and delay conditioning requires urgent dialysis. This study aimed to explore the experience of urgent dialysis patients with end-stage renal disease.<br>Methods<br>This study used a purposive sampling strategy with semi-structured interviews leading to in-depth interviews. Patients were recruited from the nephrology ward and hemodialysis center of a northern Taiwanese hospital. All participants were aged over 20 years with end-stage renal disease. Although advised by doctors to undergo dialysis, these patients delayed their treatment and later suffered severe complications. After emergency hospitalization, the patients' condition improved. Data were analyzed by content analysis.<br>Results<br>Interviews with five participants suffering from end-stage renal disease identified six themes: "experiencing a sudden jolt," "silent organ brings the most pain," "feeling angry: why me?," "facing a dilemma," "taking risks," and "facing consequences."<br>Conclusion<br>These patients delayed their treatment and later suffered severe complications, even though doctors advised them to undergo dialysis. Health professionals play an important role in communication and coordination, assisting patients in coping with their situation. The analysis of the reasons for the delay in undergoing dialysis, therefore, should help health professionals provide proper guidance and care to patients who are faced with the decision to accept dialysis treatment.
**Order of Authors:**	Min-Ling Lin
	KUEI-HUI CHU, PhD
**Response to Reviewers:**	We have modified the manuscript, which is now entitled "The experience of urgent dialysis patients with end-stage renal disease: a qualitative study" (PONE-D-21-04435), according to your comments. Our revisions are highlighted in red in the manuscript, and our modifications are detailed below. Thank you very much for reviewing our manuscript. We look forward to hearing from you.<br>Sincerely,
**Additional Information:**	
**Question**	**Response**
**Financial Disclosure**<br><br>Enter a financial disclosure statement that describes the sources of funding for the	Initials of the authors who received each award:Kuei Hui Chu<br>Source of Funding<br>This study was funded by the Taiwan Nurses Association [grant no 1062002].<br>URL of each funder website:

Powered by Editorial Manager® and ProduXion Manager® from Aries Systems Corporation

work included in this submission. Review the submission guidelines for detailed requirements. View published research articles from *PLOS ONE* for specific examples.<br><br>This statement is required for submission and **will appear in the published article** if the submission is accepted. Please make sure it is accurate.<br><br>**Unfunded studies**<br>Enter: *The author(s) received no specific funding for this work.*<br><br>**Funded studies**<br>Enter a statement with the following details:<br>• Initials of the authors who received each award<br>• Grant numbers awarded to each author<br>• The full name of each funder<br>• URL of each funder website<br>• Did the sponsors or funders play any role in the study design, data collection and analysis, decision to publish, or preparation of the manuscript?<br>• **NO** - Include this sentence at the end of your statement: *The funders had no role in study design, data collection and analysis, decision to publish, or preparation of the manuscript.*<br>• **YES** - Specify the role(s) played.<br><br>* typeset	https://www.twna.org.tw/frontend/un10_open/welcome.asp<br>The funders had no role in study design, data collection and analysis, decision to publish, or preparation of the manuscript.
**Competing Interests**<br><br>Use the instructions below to enter a competing interest statement for this submission. On behalf of all authors, disclose any competing interests that could be perceived to bias this work—acknowledging all financial support and any other relevant financial or non-financial competing interests.<br><br>This statement is required for submission and **will appear in the published article** if the submission is accepted. Please make sure it is accurate and that any funding sources listed in your Funding Information	The authors have declared that no competing interests exist.

Powered by Editorial Manager® and ProduXion Manager® from Aries Systems Corporation

later in the submission form are also declared in your Financial Disclosure statement.<br><br>View published research articles from *PLOS ONE* for specific examples.<br><br>**NO authors have competing interests**<br><br>Enter: *The authors have declared that no competing interests exist.*<br><br>**Authors with competing interests**<br><br>Enter competing interest details beginning with this statement:<br><br>*I have read the journal's policy and the authors of this manuscript have the following competing interests: [insert competing interests here]*<br><br>* typeset	
**Ethics Statement**<br><br>Enter an ethics statement for this submission. This statement is required if the study involved:<br><br>• Human participants<br>• Human specimens or tissue<br>• Vertebrate animals or cephalopods<br>• Vertebrate embryos or tissues<br>• Field research<br><br>Write "N/A" if the submission does not require an ethics statement.<br><br>General guidance is provided below. Consult the submission guidelines for detailed instructions. **Make sure that all information entered here is included in the Methods section of the manuscript.**	Institutional Review Board of the Cathay General Hospital<br>The study was approved by the appropriate institutional review board (IRB-CGH-LP 105009).

**Format for specific study types**<br><br>**Human Subject Research (involving human participants and/or tissue)**<br>• Give the name of the institutional review board or ethics committee that approved the study<br>• Include the approval number and/or a statement indicating approval of this research<br>• Indicate the form of consent obtained (written/oral) or the reason that consent was not obtained (e.g. the data were analyzed anonymously)<br><br>**Animal Research (involving vertebrate animals, embryos or tissues)**<br>• Provide the name of the Institutional Animal Care and Use Committee (IACUC) or other relevant ethics board that reviewed the study protocol, and indicate whether they approved this research or granted a formal waiver of ethical approval<br>• Include an approval number if one was obtained<br>• If the study involved *non-human primates*, add *additional details* about animal welfare and steps taken to ameliorate suffering<br>• If anesthesia, euthanasia, or any kind of animal sacrifice is part of the study, include briefly which substances and/or methods were applied<br><br>**Field Research**<br><br>Include the following details if this study involves the collection of plant, animal, or other materials from a natural setting:<br>• Field permit number<br>• Name of the institution or relevant body that granted permission	
**Data Availability**<br><br>Authors are required to make all data underlying the findings described fully available, without restriction, and from the time of publication. PLOS allows rare exceptions to address legal and ethical concerns. See the PLOS Data Policy and FAQ for detailed information.	Yes - all data are fully available without restriction

A Data Availability Statement describing where the data can be found is required at submission. Your answers to this question constitute the Data Availability Statement and **will be published in the article**, if accepted.<br><br>**Important:** Stating 'data available on request from the author' is not sufficient. If your data are only available upon request, select 'No' for the first question and explain your exceptional situation in the text box.<br><br>Do the authors confirm that all data underlying the findings described in their manuscript are fully available without restriction?	
**Describe where the data may be found in full sentences. If you are copying our sample text, replace any instances of XXX with the appropriate details.**<br><br>• If the data are **held or will be held in a public repository**, include URLs, accession numbers or DOIs. If this information will only be available after acceptance, indicate this by ticking the box below. For example: *All XXX files are available from the XXX database (accession number(s) XXX, XXX.*).<br>• If the data are all contained **within the manuscript and/or Supporting Information files**, enter the following: *All relevant data are within the manuscript and its Supporting Information files.*<br>• If neither of these applies but you are able to provide **details of access elsewhere**, with or without limitations, please do so. For example:<br><br>*Data cannot be shared publicly because of [XXX]. Data are available from the XXX Institutional Data Access / Ethics Committee (contact via XXX) for researchers who meet the criteria for access to confidential data.*<br><br>*The data underlying the results presented in the study are available from (include the name of the third party*	All relevant data are within the manuscript and its Supporting Information files.

and contact information or URL).<br>• This text is appropriate if the data are owned by a third party and authors do not have permission to share the data.<br><br>* typeset	
Additional data availability information:	Tick here if the URLs/accession numbers/DOIs will be available only after acceptance of the manuscript for publication so that we can ensure their inclusion before publication.

Powered by Editorial Manager® and ProduXion Manager® from Aries Systems Corporation

Supporting Information Click here to access/download;Manuscript;Title Page.docx

The experience of urgent dialysis patients with end-stage renal disease: a qualitative study

Running title: Mentality of patients delaying dialysis therapy

Min-Ling Lin[1], Kuei-Hui Chu[2*]

[1]MSN, RN, Head Nurse, Mackay Memorial Hospital, School of Nursing, National Yang-Ming University, Taipei, Taiwan.

[2]PhD, RN, Assistant Professor, Department of Nursing, Ching Kuo Institute of Management and Health, Keelung, Taiwan.

[*]**Corresponding Author**: Kuei-Hui Chu

Assistant Professor, Department of Nursing, Ching Kuo Institute of Management and Health, Keelung, No. 336 Fu-Hsing Rd., Keelung, 20301, Taiwan, R.O.C.

Phone: 886-2-24372093

Fax: 886-2-24376209

E-mail: kueihuichu@gmail.com

Conflicts of interest

The authors declare that there are no conflicts of interest.

Source of Funding

This study was funded by the Taiwan Nurses Association [grant no 1062002].

Authors' Contributions

Study conception and design: Min-Ling Lin, Kuei-Hui Chu

Data collection: Kuei-Hui Chu

Data analysis and interpretation: Min-Ling Lin, Kuei-Hui Chu

Drafting of the article: Min-Ling Lin, Kuei-Hui Chu

Critical revision of the article: Kuei-Hui Chu

Manuscript Click here to access/download;Manuscript;Manuscript.docx

The experience of urgent dialysis patients with end-stage renal disease: a qualitative study

ABSTRACT

Purpose

Taiwan is among the countries with the highest global prevalence of chronic renal disease. However, when advised to undergo dialysis therapy, patients with end-stage renal disease often hesitate. Attitudes toward medication and Taiwanese cultures are the main reasons for this delay, and delay conditioning requires urgent dialysis. This study aimed to explore the experience of urgent dialysis patients with end-stage renal disease.

Methods

This study used a purposive sampling strategy with semi-structured interviews leading to in-depth interviews. Patients were recruited from the nephrology ward and hemodialysis center of a northern Taiwanese hospital. All participants were aged over 20 years with end-stage renal disease. Although advised by doctors to undergo dialysis, these patients delayed their treatment and later suffered severe complications. After emergency hospitalization, the patients' condition improved. Data were analyzed by content analysis.

Results

Interviews with five participants suffering from end-stage renal disease identified six themes: "experiencing a sudden jolt," "silent organ brings the most pain," "feeling angry: why me?," "facing a dilemma," "taking risks," and "facing consequences."

Conclusion

These patients delayed their treatment and later suffered severe complications, even though doctors advised them to undergo dialysis. Health professionals play an important role in communication and coordination, assisting patients in coping with their situation. The analysis of the reasons for the delay in undergoing dialysis, therefore, should help health professionals provide proper guidance and care to patients who are faced with the decision to accept dialysis treatment.

KEYWORDS: End-Stage Renal Disease, Hemodialysis, Qualitative Research

INTRODUCTION

In 2019, renal diseases, including nephritis, nephrotic syndrome, and nephropathy, were among the top 10 causes of mortality in Taiwan [1]. In 2016, the annual United States Renal Data System [2] reported that Taiwan had the highest reported prevalence rate of dialysis. Improper medication and the use of alternative treatments that are not thoroughly examined cause patients with end-stage renal disease (ESRD) to develop uremia within a short period of time [3]. When patients with ESRD are informed on the need for kidney transplantation or dialysis to improve quality of life, they tend to opt for alternative therapy [4].

In the early stages of chronic kidney disease (CKD), patients experience few signs or symptoms. Owing to the lack of knowledge about the disease and preference for folk or herbal medicine, patients with CKD can miss out on the benefits of appropriate early treatment. When it leads to irreversible renal failure (end-stage renal disease), only dialysis treatment can reduce the symptoms of the disease. If not treated by dialysis, patients may suffer from acute respiratory failure, metabolic acidosis, or other severe complications, delaying treatment and even causing death [5]. Some patients hesitate to act when faced with life-changing diseases [6-8]. Many patients experience uncertainty, fear, sadness, or anger [9] and refuse or delay starting dialysis [10] until they are forced to accept treatment after the development of life-threatening complications such as acute pulmonary edema and heart and respiratory failure [11]. This not only raises mortality rates but also causes worry for patients' families and

increases medical costs [12-14].

Before the diagnosis of ESRD, most patients have preserved renal function. However, the feeling of helplessness when told of the need for dialysis and hesitancy to start dialysis often worsen their condition. When symptoms become life-threatening, patients will be arranged for urgent admission to the hospital for urgent dialysis. Therefore, this study aimed to explore the experience of urgent dialysis patients with end-stage renal disease.

METHODS

Research Design

This study used a qualitative design with purposive sampling to collect data. In-depth interview questions were designed in accordance with related literature, such as illness representations and coping processes of patients [4]. Data were collected between October and December 2016.

Participants/Setting

The study recruited participants from the nephrology ward of a hospital in northern Taiwan. Participants were aged over 20 years and were diagnosed with ESRD accompanied by uremia. The characteristics of the study participants are summarized in Table 1. All five patients were diagnosed with ESRD. Although their doctors had recommended dialysis, these patients delayed treatment, resulting in severe complications (acute respiratory failure, acute pulmonary edema, hyperkalemia, cardiac arrhythmia, and heart failure) and admission to the intensive care unit. Later, these patients were hospitalized and underwent emergency hemodialysis. All the

patients were conscious with no hearing difficulties and agreed to the interviews. Patients who had hemodialysis vascular access and were scheduled for treatment (AV-shunt and Hickman catheter) were excluded. The sample size was determined when data saturation was achieved. In other words, the researcher analyzed the information from the samples until no new information could be obtained.

Data Collection and Analysis

The study was approved by the appropriate institutional review board (IRB-CGH-LP 105009). After explaining the purpose of the study to the participants, the researcher received written informed consent and made appointments for interviews. Interviews were conducted in environments where the participants felt most comfortable answering questions. The interviews were conducted privately, and participants were assured that their personal information would be kept confidential. The interviews were recorded and transcribed for further analysis and interpretation. All conversations took approximately 30 minutes.

Instrument

A semi-structured interview guide was used for the in-depth interviews (Table 2). In line with the study's goal, the interviews aimed to explore the patients' mentality and understand the experience of urgent dialysis. The instruments used included the researcher's experiences, the interview guide, and a recorder to collect data during the interviews. Therefore, the researcher was the major instrument used in this study. After data collection and analysis, data was

described consistently to meet the needs of dependability. Coding was conducted for data analysis and conceptualization.

RESULTS

From the results, six major themes were identified: “experiencing a sudden jolt,” “silent organ brings the most pain,” “feeling angry: why me?,” “facing a dilemma,” “taking risks,” and “facing consequences.” These are described below and summarized in Table 3.

Experiencing a sudden jolt

At first, the patients did not experience any symptoms and were referred to the nephrology department from the emergency room. After interacting with the doctor, they became aware of their illness. Subsequently, feeling as though they had received a life sentence, they became deeply frustrated. After the diagnosis, they sensed that their lives would completely change and felt sorry for themselves. Participant A: *“I was transferred to the nephrology department and underwent renal ultrasound, which showed renal atrophy.”* Participant E: *“I had been suffering from leg cramps. So, it was recommended that I undergo a check-up at the hospital, where I was told that one of my kidneys was not functioning properly.”*

Silent Organ Brings the Most Pain

The kidneys are regarded as silent organs; there are no obvious symptoms of deteriorating function, causing the condition to be ignored. A diagnosis of kidney failure is made only when patients are aware of symptoms that consistently lower their quality of life. Participant A:

"Whenever the weather changed, I felt uncomfortable." Participant C: *"I had edema and general discomfort, and I felt shortness of breath during work."*

Feeling Angry: Why Me?

Participants who were recommended to undergo dialysis and prepare for vascular access felt furious and were unable to accept the fact, thereby attempting to avoid treatment. Participant B: *"I thought I should just procrastinate and keep working as dialysis would not cure my illness."* Participant D: *"I felt I would rather die than undergo dialysis."*

Facing a Dilemma

As kidney failure has no cure, participants hesitated when faced with the decision to undergo hemodialysis. Feeling uncomfortable, they could not make up their minds to accept the treatment. They felt like they were going back and forth on the decision and did not know where to go. In the interviews, participants expressed their sadness and disappointment. Participant A: *"I tried alternative treatment and religion. Neither was successful. I felt that if I accepted hemodialysis, it would take away my time and I would not be able to work."* Participant B: *"I asked around and most people said it would not help. I tried Chinese medicine and a different diet, but they were not effective. What was I to do? I did not want to undergo dialysis. I was worried and annoyed."* Participant C: *"I felt that if I underwent* hemodialysis, *I would have to spend a lot of time at the hospital, leaving me unable to do my job (farming). I needed to feed my wife and kids, but I was out of breath and unable to work. That was annoying."*

Taking Risks

While the participants hesitated to undergo dialysis, their condition became unbearable. At this stage, family, friends, and health professionals patiently supported and listened to them. Eventually, participants would start treatment. Participant B: *"I was worried that the burn wound on my left hand would be infected and I would end up having to undergo amputation, so I finally agreed to dialysis. My family had always advised me to start dialysis.*" Participant D: *"My body has become like this. What could I do? I had no choice."*

Facing Consequences

Without dialysis, participants suffered all kinds of pain, with their families experiencing mental stress. Participant B: *"I still do not want to do it. People die, stop pursuing me."* Participant C: *"Dialysis goes on forever, and I will not be able to work. I would rather die."*

DISCUSSION

This study aimed at exploring the experience of urgent dialysis patients with end-stage renal disease. Six themes were identified in this study, which included "experiencing a sudden jolt," "silent organ brings the most pain,", "feeling angry: why me?", "facing a dilemma," "taking risks," and "facing consequences." This result is similar to those of previous studies [15, 14]. However, among all the factors causing delay in dialysis therapy, this study identified facing a dilemma, which included family finances, to be the main reason. In the case of hemodialysis,

patients are usually connected to a machine for three to four hours, three days a week, in a dialysis center and thus, their daily lives may be greatly affected [6, 13]. The participants experienced physical and mental challenges due to renal disease; all of them had experienced anxiety, sadness, denial, depression, low self-esteem, and helplessness at some point [16]. Therefore, they had trouble accepting their condition and refused or delayed dialysis therapy. Their reactions were consistent with those found in previous studies [14, 15].

Contextual information also provided findings similar to those of previous studies. Participants in this study sought alternative therapy when considering the possible side effects of dialysis and renal failure [8]. Another finding was that three out of five participants cited financial issues as the main reason for delaying dialysis therapy, which led to urgent dialysis. As these participants were the main breadwinners of their families, they were worried that undergoing dialysis therapy two to three times a week would interfere with their work, thereby affecting their earning potential. Therefore, it would be helpful if the government's social welfare department could issue disability cards and provide patients with access to social workers.

Patients and their families need an outlet to express their sorrow and anger. Considering these needs, health professionals must attempt to resolve their doubts and help them understand the treatment process. Previous research has demonstrated that the information presented to patients is often incomplete and difficult to grasp [17]. Providing accurate information and pre-

dialysis education is essential in empowering patients with ESRD to choose dialysis modalities [6]. A video introduction to the dialysis process and environment could reduce patients' concerns. Health professionals could also refer patients to support groups and organizations that offer subsidies to help alleviate their economic burden. The goal should be to help patients cope with long-term dialysis therapy.

This study had a few limitations. First, the age range of the participants in this study was quite broad. As different age groups may face different kinds of stress, the results of this study are generalizable only to patients with ESRD from similar age groups. Therefore, future studies must recruit patients of varying ages. Another limitation was that the participants in this study were recruited from a specific regional hospital. Thus, it is not certain if the findings can be generalized to patients with ESRD from other regions.

During the interviews, the researcher tried to view the situation from the participants' perspective and understand their fear and hesitation with regard to dialysis therapy. From the interviews, it was evident that the patients needed companionship and someone to lend an ear. A trusting relationship with health professionals could help them accept proper treatment. Health professionals should, therefore, be willing to provide mental support and companionship to patients with ESRD.

CONCLUSION

Patients with ESRD have mixed feelings about dialysis therapy and focus on their negative emotions. Therefore, health professionals should play an important role by communicating and coordinating with patients to help them cope with their situation, and make them feel confident to accept proper treatment.

References

1. Ministry of Health and Welfare [Internet]. Death cause statistics; 2019 [cited 2021 February 9]. Available from: https://www.mohw.gov.tw/cp-3795-41794-1.html 6.15.
2. United States Renal Data System [Internet]. End-stage renal disease in the United States; 2016 [cited 2019 Apr 13]. Available from: https://www.usrds.org/2016/view/Default.aspx.
3. Wu MY, Wu MS. Taiwan renal care system: A learning health-care system. Nephrology. 2018;23(S4):112.115. https://doi.org/10.1111/nep.13460
4. Lin CC, Chen MC, Hsieh HF, Chang SC. Illness representations and coping processes of Taiwanese patients with early-stage chronic kidney disease. Journal of Nursing Research. 2013;21(2):120.128. https://doi.org/10.1097/jnr.0b013e3182921fb8
5. Wu CY. A nursing experience of applying the health belief model to an emergency patient who refused acute hemodialysis. The Kaohsiung Journal of Nursing. 2016;33(1):53.62. https://DOI:10.6692/KJN-2016-33-1-6
6. Chen NH, Lin YP, Liang SY, Tung HH, Tsay SL, Wang TJ. Conflict when making decisions about dialysis modality. Journal of Clinical Nursing. 2018;27(1-2):e138.e146. https://doi.org/10.1111/jocn.13890
7. McPherson L, Basu M, Gander J, Pastan SO, Mohan S, Wolf MS, et al. Decisional conflict between treatment options among end-stage renal disease patients evaluated for

kidney transplantation. Clinical Transplantation. 2017;31(7):e12991. https://doi.org/10.1111/ctr.12991

8. Shayeb ME, Scarfe A, Yasui Y, Winget M. Reasons physicians do not recommend and patients refuse adjuvant chemotherapy for stage III colon cancer: A population-based chart review. BMC Research Notes. 2012;5(269). https://doi.org/10.1186/1756-0500-5-269

9. Yu IC, Tsai YF. From silence to storm--patient illness trajectory from diabetes diagnosis to haemodialysis in Taiwan: a qualitative study of patients' perceptions. Journal of Advanced Nursing. 2013;69(9):1943.1952. https://doi.org/10.1111/jan.12055.

10. Sellars M, Clayton JM, Morton RL, Luckett T, Silvester W, Spencer L, et al. An interview study of patient and caregiver perspectives on advance care planning in ESRD. American Journal of Kidney Diseases. 2018;71(2):216.224. https://doi.org/10.1053/j.ajkd.2017.07.021

11. Lin WH, Kuo KL, Hsia CC, Wu TY, Chen CC et al. Overview of chronic kidney disease. Taipei City Medical Journal. 2013;10(2):101.109. https://doi.org/10.6200/TCMJ.2013.10.2.01

12. Chang YK, Hsu CC, Chen PC, Chen YS, Hwang SJ, Li TC, et al. Trends of cost and mortality of patients on haemodialysis with end stage renal disease. Nephrology. 2015;20(4):243.249. https://doi.org/10.1111/nep.12380

13. Tchape ODM, Tchapoga YB, Atuhaire C, Priebe G, Cumber SN. Physiological and psychosocial stressors among hemodialysis patients in the Buea Regional Hospital, Cameroon. The Pan African Medical Journal. 2018;30(49). https://doi.org/10.11604/pamj.2018.30.49.15180

14. Yang YC, Siao YD (2013). Nursing care experience of a end-stage renal disease on long-term hemodialysis. Journal of Taiwan Nephrology Nurses Association. 2013;12(1):87.98. https://www.airitilibrary.com/Publication/alDetailedMesh?docid=17267404-201303-201305230034-201305230034-72-86

15. Chen M K, Lin YS. The voices from the Remission Society: Exploring the narrative of illness experience and psychological adaptation process in patients with renal failure. The Journal of Kaohsiung Behavioural Sciences. 2014; 4:29.55. http://www.airitilibrary.com/Publication/Index/P20130902001-201405-201407020008-201407020008-29-55

16. Chiang HH, Livneh H, Yen ML, Li TS, Tsai TY. Prevalence and correlates of depression among chronic kidney disease patients in Taiwan. BMC Nephrology. 2013;14(78). https://doi.org/10.1186/1471-2369-14-78.

17. Prieto- Velasco M, Quiros P, Remon C. The concordance between patients' renal replacement therapy choice and definitive modality: Is it a utopia? PLoS One.

2015;10(10):e0138811. https://doi.org/10.1371/journal.pone.0138811

TABLES

Table 1. Characteristics of the study participants (N = 5)

	**Sex**	**Age**	**Education Level**	**Marital Status**	**Work status**	**History**	**Diagnosis**
A	Male	62	secondary	Widowed	Yes	Hypertension	ESRD
B	Male	60	Elementary school	Yes	Yes	Hypertension	ESRD
C	Male	78	Elementary school	No	No	DM, Hypertension	ESRD
D	Female	72	illiterate	Yes	No	Hypertension, DM, Heart disease	ESRD
E	Male	78	Elementary school	No	No	DM, Hypertension	ESRD

Table 2. Semi-structured Interview Guide used for the In-depth Interviews

1. Can you describe the disease process?
2. What was the condition for this medical treatment?
3. What kind of treatment method did the doctor suggest?
4. How are you feeling right now?
5. Do you accept the treatment method recommended by the doctor? (1) Yes (2) No (why?)
6. What did you do when you declined the treatment?
7. What is the reason for visiting the hospital this time? What symptoms do you have?
8. Can you describe your feeling after the first dialysis experience?

Table 3. Six major themes identified in the study

Themes
Experiencing a sudden jolt
Silent organ brings the most pain
Feeling angry: why me?
Facing a dilemma
Taking risks
Facing consequences

Click here to access/download
Supporting Information
Characteristics of the study participants.pdf

Click here to access/download
Supporting Information
IRB Approval.pdf

The experience of urgent dialysis patients with end-stage renal disease: a qualitative study

Running title: Mentality of patients delaying dialysis therapy

Min-Ling Lin[1], Kuei-Hui Chu[2*]

[1]MSN, RN, Head Nurse, Mackay Memorial Hospital, School of Nursing, National Yang-Ming University, Taipei, Taiwan.

[2]PhD, RN, Assistant Professor, Department of Nursing, Ching Kuo Institute of Management and Health, Keelung, Taiwan.

[*]Corresponding Author: Kuei-Hui Chu

Assistant Professor, Department of Nursing, Ching Kuo Institute of Management and Health, Keelung, No. 336 Fu-Hsing Rd., Keelung, 20301, Taiwan, R.O.C.

Phone: 886-2-24372093

Fax: 886-2-24376209

E-mail: kueihuichu@gmail.com

Conflicts of interest

The authors declare that there are no conflicts of interest.

Source of Funding

This study was funded by the Taiwan Nurses Association [grant no 1062002].

Authors' Contributions

Study conception and design: Min-Ling Lin, Kuei-Hui Chu

Data collection: Kuei-Hui Chu

Data analysis and interpretation: Min-Ling Lin, Kuei-Hui Chu

Drafting of the article: Min-Ling Lin, Kuei-Hui Chu

Critical revision of the article: Kuei-Hui Chu

INTRODUCTION

In 2019, renal diseases, including nephritis, nephrotic syndrome, and nephropathy, were among the top 10 causes of mortality in Taiwan [1]. In 2016, the annual United States Renal Data System [2] reported that Taiwan had the highest reported prevalence rate of dialysis. Improper medication and the use of alternative treatments that are not thoroughly examined cause patients with end-stage renal disease (ESRD) to develop uremia within a short period [3]. When patients with ESRD are informed of kidney transplantation or dialysis to improve their quality of life, they tend to opt for alternative therapy [4].

In the early stages of chronic kidney disease (CKD), patients experience a few signs or symptoms. Owing to the lack of knowledge about the disease and preference for folk or herbal medicine, patients with CKD can miss out on the benefits of appropriate early

treatment. When it leads to irreversible renal failure (end-stage renal disease), only dialysis treatment can reduce the disease's symptoms. If not treated by dialysis, patients may suffer from acute respiratory failure, metabolic acidosis, or other severe complications, delaying treatment and even causing death [5]. Some patients hesitate to act when faced with life-changing diseases [6-8]. Many patients experience uncertainty, fear, sadness, or anger [9] and refuse or delay starting dialysis [10] until they are forced to accept treatment after the development of life-threatening complications, such as acute pulmonary edema and heart and respiratory failure [11]. This increases mortality rates and medical costs and causes worry for patients' families [12-14].

Before the diagnosis of ESRD, most patients have preserved renal function. However, the feeling of helplessness when informed of the need for dialysis and hesitancy to start dialysis often worsen their condition. When symptoms become life-threatening, patients are scheduled for urgent admission to the hospital for urgent dialysis. Therefore, this study aimed to explore the experience of urgent dialysis patients, aged 60 years old or over, with ESRD.

Commented [A1]: It would be more precise to define age group of chosen patients.

METHODS

Research Design

This study used a qualitative design with purposive sampling to collect data. In-depth interview questions were designed according to related literature, such as illness representations and coping processes of patients [4]. Data were collected between October and December 2016.

Participants/Setting

The study recruited participants from the nephrology ward of a hospital in northern Taiwan. Participants were aged 60 years old or over and were diagnosed with ESRD accompanied by uremia. The patients had never undergone hemodialysis and were advised by the doctor to undergo this procedure. However, the patients refused and were scheduled for emergency hospitalization after slipping into coma. Afterward, the patients were transferred to the ward in a stable condition after undergoing emergency hemodialysis. The characteristics of the study participants are summarized in Table 1. All five patients were diagnosed with ESRD. Although their doctors had recommended dialysis, these patients delayed treatment, resulting in severe complications (acute respiratory failure, acute pulmonary edema, hyperkalemia, cardiac arrhythmia, and heart failure) and admission to the intensive care unit. Later, these patients were

Commented [A2]: You need to have a clear and precise inclusion criterion to inform the reader who were selected and considered eligible to be included. The information provided about sample is broad and not indicate any specification to the selected participants.

hospitalized and underwent emergency hemodialysis. All the patients were conscious with no hearing difficulties and agreed to the interviews.

Patients who had hemodialysis vascular access and were scheduled for treatment (AV-shunt and Hickman catheter) were excluded because catheter insertion was indicated in these cases due to the decision to undergo hemodialysis. The sample size was determined when data saturation was achieved. In other words, the researcher analyzed the information from the samples until no new information could be obtained.

Data Collection and Analysis

The study was approved by the appropriate Institutional Review Board (IRB-CGH-LP 105009). Eligible cases were referred by a physician, and face-to-face interviews were conducted for each patient by two trained researchers in the hospital ward. After explaining the study's purpose to the participants, the researcher received written informed consent and scheduled appointments for interviews. Interviews were conducted in environments where the participants felt most comfortable answering questions. The interviews were conducted privately, and participants were assured that their personal information would be kept confidential. The interviews were recorded

Commented [A3]: Mention why!

Commented [A4]: You should specify which approach did you follow in analysing data as there are different methods. There are two general types of content analysis: conceptual analysis and relational analysis. Conceptual analysis determines the existence and frequency of concepts in a text. Relational analysis develops the conceptual analysis further by examining the relationships among concepts in a text. Each type of analysis may lead to different results, conclusions, interpretations, and meanings.

Commented [A5]: Where? Again, this is a short cut information. The reader needs to have detailed information about setting and exactly how the participants were approached and interviewed. Who conducted the interview, were all the interviews conducted at the same time and day by the same researcher etc.. more details are needed please.

Commented [A6]: Not sure what does that mean ... do you mean it was an individual interview to each patient please clarify need more details?

and transcribed for further analysis and interpretation. All conversations took approximately 30 minutes. The interview commenced with the description of the disease process and continued following the interview guide. Data collected in approximately two months were used for conceptual content analysis.

The interview content was recorded using audio recordings, and the data were analyzed and summarized according to the content analysis method of Miles and Huberman (1994). The analysis steps were as follows:

1. The recorded contents of the participants were transcribed verbatim.

2. The recorded texts were carefully read, and the sentences whose meaning were confirmed were described.

3. Meaningful sentences were categorized according to content or create categories.

4. The relationship between categories and themes was categorized and summarized to form themes.

Instrument

A semi-structured interview guide was used for the in-depth interviews (Table 2). According to the study's goal, the interviews aimed to comprehensively investigate the patients' perceptions or beliefs and explore cases of urgent dialysis. The instruments used included the researcher's experiences, the interview guide, and a recorder to collect data during the interviews. Therefore, based on the literature, the researcher developed

Commented [A7]: You mean with all participants or do you mean each individual interview. Also, you need to mention how was the interview initiated and continued give details please

Commented [A8]: I would suggest using another expression such as deep investigation about perception or beliefs etc

Commented [A9]: Not sure what was mean this is not correctly written?? You might mean that the researcher developed the question which was used to lead the interview and to draw the data. If this was the case you need to mention in details how was the questions were formulated and from where did you draw the them was it from the literature or from similar r study etc … we never say that the researcher was the tool????

Commented [A10R9]:

questions to conduct the interview and draw data. After data collection and analysis, data were described consistently to meet the needs of dependability. Coding was conducted for data analysis and conceptualization.

RESULTS

The following six organizing themes were revealed in the urgent dialysis patients, aged 60 years old or over, with ESRD: "experiencing a sudden jolt," "silent organ brings the most pain," "feeling angry: why me?" "facing a dilemma," "taking risks," and "facing the consequences." These are described below and summarized in Table 3.

Experiencing a sudden jolt

At first, the patients did not experience any symptoms and were referred to the emergency room's nephrology department. After interacting with the doctor, they became aware of their illness. Subsequently, feeling as though they had received a life sentence, they became deeply frustrated. After the diagnosis, they sensed that their lives would completely change and felt sorry for themselves.

Participant A: *"I was transferred to the nephrology department and underwent renal ultrasound, which showed renal atrophy."*

Suddenly, the patients developed symptoms that were not related to the kidneys; however, they were diagnosed of kidney dysfunction, which surprised them.

Commented [A11]: Similar study is unnecessary in this context; the term literature encompasses all previous studies.

Commented [A12]: Method of data collection and analysis are missed and detailed describiton are needed to be added to this section. The method used for data analysis should be scientifecly described even if it was a qualtive study .. there are several methods to analyse the data to draw the themes of you study. It is a big fall of ther study if you did not follow one of them.
One example is Melis method of content analysis another one is Roger's method so you should mention clearly which method did you follow and explain it in details

Commented [A13]: Before mentioned the major themes there is a need to explain the word, phrases and then themes and subthemes found in the study this is a very short summary of your findings.

Commented [A14]: General steps for conducting a conceptual content analysis:
1. Decide the level of analysis: word, word sense, phrase, sentence, themes.
2. Decide how many concepts to code for: develop pre-defined or interactive set of categories or concepts. Decide either: A. to allow flexibility to add categories through the coding process, or B. to stick with the pre-defined set of categories.

Commented [A15]: What was the subthemes found under this major findings?

Participant E: *"I had been suffering from leg cramps. So, it was recommended that I undergo a check-up at the hospital, where I was told that one of my kidneys was not functioning properly."*

Silent Organ Brings the Most Pain

Commented [A16]: The same for this one see above notes

The kidneys are regarded as silent organs; there are no obvious symptoms indicating deteriorating function, causing the condition to be ignored. A diagnosis of kidney failure is established only when patients are aware of symptoms that consistently lower their quality of life.

Participant A: *"Whenever the weather changed, I felt uncomfortable."*

The symptoms had obviously affected the patients' life and made them feel very uncomfortable.

Participant C: *"I had edema and general discomfort, and I felt shortness of breath during work."*

Feeling Angry: Why Me?

Commented [A17]: The same comments see above please.

Participants who were recommended to undergo dialysis and prepare for vascular access felt furious and unable to accept the fact, thereby avoiding treatment. They believed that dialysis could not completely cure the disease; rather, it would only

maintain the status.

Participant B: *"I thought I should just procrastinate and keep working as dialysis would not cure my illness."*

Participants were afraid that dialysis would affect their lives and, thus, chose to escape by refusing to accept formal treatment.

Participant D: *"I felt I would rather die than undergo dialysis."*

Facing a Dilemma

As kidney failure has no cure, participants hesitated when faced with the decision to undergo hemodialysis. Feeling uncomfortable, they could not decide to accept the treatment. They felt like they were going back and forth on the decision and did not know where to go.

Participant A: *"I tried alternative treatment and religion. Neither was successful. I felt that if I accepted hemodialysis, it would take away my time, and I would not be able to work."*

Unwilling to undergo dialysis, they searched for alternative treatments; however, the outcomes were unsatisfactory. In the interviews, participants expressed their sadness and disappointment.

Participant B: *"I asked around, and most people said it would not help. I tried Chinese*

medicine and a different diet, but they were not effective. What was I to do? I did not want to undergo dialysis. I was worried and annoyed."

Economic factors prevented participants from receiving dialysis because it is time-consuming; additionally, their bodies were weak, and they could not work.

Participant C: *"I felt that if I underwent hemodialysis, I would have to spend a lot of time at the hospital, leaving me unable to do my job (farming). I needed to feed my wife and kids, but I was out of breath and unable to work. That was annoying."*

Taking Risks

While the participants hesitated to undergo dialysis, their condition became unbearable. At this stage, family, friends, and health professionals patiently supported and listened to them. Eventually, participants would start treatment, accepted with poor outcomes of delayed dialysis.

Participant B: *"I was worried that the burn wound on my left hand would be infected and I would end up having to undergo amputation, so I finally agreed to dialysis. My family had always advised me to start dialysis."*

The participants had no choice but to agree with dialysis being the only option.

Participant D: *"My body has become like this. What could I do? I had no choice."*

Facing Consequences

Without dialysis, participants suffered all kinds of pain, with their families experiencing mental stress.

Participant B: *"I still do not want to do it. People die; stop pursuing me."*

The participants felt that there is no end to dialysis after commencement and that being unable to work or take care of their families would be more painful.

Participant C: *"Dialysis goes on forever, and I will not be able to work. I would rather die."*

DISCUSSION

Commented [A18]: Need further elaboration study findings and relating it with other research findings

This study aimed at exploring the experience of urgent dialysis patients with ESRD. Six themes were identified in this study, which included "experiencing a sudden jolt," "silent organ brings the most pain," "feeling angry: why me?", "facing a dilemma," "taking risks," and "facing the consequences." This result is similar to those of previous studies [15, 14]. In particular, patients who will spend a considerable time of their lives receiving hemodialysis require constant physical and emotional support. However, among all the factors causing a delay in dialysis therapy, this study identified facing a dilemma, which included family finances, to be the main reason. In the case of

hemodialysis, patients are usually connected to a machine for three to four hours, three days a week, in a dialysis center. Thus, their daily lives may be significantly affected [6, 13]. In Taiwan, most families assume that men need to make money to pay for family expenses; thus, patients had to consider family finances.

The participants experienced physical and mental challenges due to renal disease; all of them had experienced anxiety, sadness, denial, depression, low self-esteem, and helplessness at some point [16]. Therefore, they had trouble accepting their condition and refused or delayed dialysis therapy.

Their reactions were consistent with those found in previous studies [14, 15]. Contextual information also provided findings similar to those of previous studies; hence, there is a clear need to provide holistic care for patients physically and psychologically.

Participants in this study sought alternative therapy when considering the possible side effects of dialysis and renal failure [8]. Another finding was that three out of five participants cited financial issues as the main reason for delaying dialysis therapy, which led to urgent dialysis. As these participants were the primary breadwinners of their families, they were worried that undergoing dialysis therapy two to three times a

week would interfere with their work, thereby affecting their earning potential. Therefore, it would be helpful if the government's social welfare department could issue disability cards and provide patients with access to social workers.

Patients and their families need an outlet to express their sorrow and anger. Considering these needs, health professionals should resolve their doubts and help them understand the treatment process. Previous research demonstrated that patients' information is often incomplete and difficult to grasp [17]. Providing accurate information and pre-dialysis education is essential in empowering ESRD patients to choose dialysis modalities [6]. A video introduction of the dialysis process and environment could reduce patients' concerns. Health professionals could also refer patients to support groups and organizations that offer subsidies to alleviate their economic burden. The goal should be to help patients cope with long-term dialysis therapy.

During the interviews, the researcher tried to view the situation from the participants' perspective and understand their fear and hesitation concerning dialysis therapy. From the interviews, it was evident that the patients needed companionship and someone to lend an ear. A trusting relationship with health professionals could help them accept proper treatment. Health professionals should, therefore, be willing to provide mental

support and companionship to patients with ESRD.

This study had a few limitations. First, the age range of the participants in this study was quite broad. As different age groups may face different kinds of stress, the results of this

study are generalizable only to patients with ESRD in similar age groups. Therefore, future

studies should recruit patients of varying ages. Another limitation was that the participants in this study were recruited from a specific regional hospital, which may limit the generalizability of the results. Another limitation implicit in qualitative studies is the possibility of researcher bias in interpretation. To mitigate this, three researchers were deeply involved in the data analysis.

The qualitative interviews were conducted retrospectively to retrieve experiences and thoughts. When the doctor recommends dialysis, the patient thoughts and practices or family members might have contributed different insights, and further study may, therefore, be needed to target them specifically.

CONCLUSION

Patients with ESRD have mixed feelings about dialysis therapy and focus on their negative emotions and family financial burden. Therefore, health professionals should

Commented [A19]: Need to include how the findings would help to change the current practice at selected settings and what should be done further as an evidence-based practice to improve current condition. Also, you need to mention the limitations in your study. This would guide conducting of further research.

play an essential role by communicating and coordinating with patients and their family members to help them cope with their situation and feel confident to accept proper treatment.

References

1. Ministry of Health and Welfare [Internet]. Death cause statistics; 2019 [cited 2021 February 9]. Available from: https://www.mohw.gov.tw/cp-3795-41794-1.html 6.15.
2. United States Renal Data System [Internet]. End-stage renal disease in the United States; 2016 [cited 2019 Apr 13]. Available from: https://www.usrds.org/2016/view/Default.aspx.
3. Wu MY, Wu MS. Taiwan renal care system: A learning health-care system. Nephrology. 2018;23(S4):112.115. https://doi.org/10.1111/nep.13460
4. Lin CC, Chen MC, Hsieh HF, Chang SC. Illness representations and coping processes of Taiwanese patients with early-stage chronic kidney disease. Journal of Nursing Research. 2013;21(2):120.128. https://doi.org/10.1097/jnr.0b013e3182921fb8
5. Wu CY. A nursing experience of applying the health belief model to an emergency patient who refused acute hemodialysis. The Kaohsiung Journal of Nursing. 2016;33(1):53.62. https://DOI:10.6692/KJN-2016-33-1-6

6. Chen NH, Lin YP, Liang SY, Tung HH, Tsay SL, Wang TJ. Conflict when making decisions about dialysis modality. Journal of Clinical Nursing. 2018;27(1-2):e138.e146. https://doi.org/10.1111/jocn.13890

7. McPherson L, Basu M, Gander J, Pastan SO, Mohan S, Wolf MS, et al. Decisional conflict between treatment options among end-stage renal disease patients evaluated for kidney transplantation. Clinical Transplantation. 2017;31(7):e12991. https://doi.org/10.1111/ctr.12991

8. Shayeb ME, Scarfe A, Yasui Y, Winget M. Reasons physicians do not recommend and patients refuse adjuvant chemotherapy for stage III colon cancer: A population-based chart review. BMC Research Notes. 2012;5(269). https://doi.org/10.1186/1756-0500-5-269

9. Yu IC, Tsai YF. From silence to storm--patient illness trajectory from diabetes diagnosis to haemodialysis in Taiwan: a qualitative study of patients' perceptions. Journal of Advanced Nursing. 2013;69(9):1943.1952. https://doi.org/10.1111/jan.12055.

10. Sellars M, Clayton JM, Morton RL, Luckett T, Silvester W, Spencer L, et al. An interview study of patient and caregiver perspectives on advance care planning in ESRD. American Journal of Kidney Diseases. 2018;71(2):216.224. https://doi.org/10.1053/j.ajkd.2017.07.021

11. Lin WH, Kuo KL, Hsia CC, Wu TY, Chen CC et al. Overview of chronic kidney disease. Taipei City Medical Journal. 2013;10(2):101.109. https://doi.org/10.6200/TCMJ.2013.10.2.01

12. Chang YK, Hsu CC, Chen PC, Chen YS, Hwang SJ, Li TC, et al. Trends of cost and mortality of patients on haemodialysis with end stage renal disease. Nephrology. 2015;20(4):243.249. https://doi.org/10.1111/nep.12380

13. Tchape ODM, Tchapoga YB, Atuhaire C, Priebe G, Cumber SN. Physiological and psychosocial stressors among hemodialysis patients in the Buea Regional Hospital, Cameroon. The Pan African Medical Journal. 2018;30(49). https://doi.org/10.11604/pamj.2018.30.49.15180

14. Yang YC, Siao YD (2013). Nursing care experience of a end-stage renal disease on long-term hemodialysis. Journal of Taiwan Nephrology Nurses Association. 2013;12(1):87.98. https://www.airitilibrary.com/Publication/alDetailedMesh?docid=17267404-201303-201305230034-201305230034-72-86

15. Chen M K, Lin YS. The voices from the Remission Society: Exploring the narrative of illness experience and psychological adaptation process in patients with renal failure. The Journal of Kaohsiung Behavioural Sciences. 2014; 4:29.55. http://www.airitilibrary.com/Publication/Index/P20130902001-201405-

201407020008-201407020008-29-55

16. Chiang HH, Livneh H, Yen ML, Li TS, Tsai TY. Prevalence and correlates of depression among chronic kidney disease patients in Taiwan. BMC Nephrology. 2013;14(78). https://doi.org/10.1186/1471-2369-14-78.

17. Prieto-Velasco M, Quiros P, Remon C. The concordance between patients' renal replacement therapy choice and definitive modality: Is it a utopia? PLoS One. 2015;10(10):e0138811. https://doi.org/10.1371/journal.pone.0138811

TABLES

Table 1. Characteristics of the study participants (N = 5)

	Sex	Age	Education Level	Marital Status	Work status	History	Diagnosis
A	Male	62	secondary	Widowed	Yes	Hypertension	ESRD
B	Male	60	Elementary school	Yes	Yes	Hypertension	ESRD
C	Male	78	Elementary school	No	No	DM, Hypertension	ESRD
D	Female	72	illiterate	Yes	No	Hypertension, DM, Heart disease	ESRD
E	Male	78	Elementary school	No	No	DM, Hypertension	ESRD

Table 2. Semi-structured Interview Guide used for the In-depth Interviews

1. Can you describe the disease process?
2. What was the condition for this medical treatment?
3. What kind of treatment method did the doctor suggest?
4. How are you feeling right now?
5. Do you accept the treatment method recommended by the doctor? (1) Yes (2) No (why?)
6. What did you do when you declined the treatment?
7. What is the reason for visiting the hospital this time? What symptoms do you have?
8. Can you describe your feeling after the first dialysis experience?

Table 3. Six major themes identified in the study

Themes
Experiencing a sudden jolt
Silent organ brings the most pain
Feeling angry: why me?
Facing a dilemma
Taking risks
Facing consequences

Dear Editor:

We have modified the manuscript, which is now entitled “The experience of urgent dialysis patients with end-stage renal disease: a qualitative study” (PONE-D-21-04435), according to your comments. Our revisions are highlighted in red in the manuscript, and our modifications are detailed below. Thank you very much for reviewing our manuscript. We look forward to hearing from you.

Sincerely,

Kuei-Hui Chu

Reply to the reviewers’ comments:

1. It would be more precise to define age group of chosen patients.

Reply: Accordingly, relevant descriptions have been added to the INTRODUCTION and METHODS (page 2). The inclusion criterion was patients aged 60 years old or over.

2. You need to have a clear and precise inclusion criterion to inform the reader who were selected and considered eligible to be included. The information provided about sample is broad and not indicate any specification to the selected participants.

Reply: Accordingly, relevant descriptions have been added to the Participants/Setting subsection of the METHODS (page 2). We have added a clear and precise inclusion criterion and included details regarding age and disease characteristics.

3. “Patients who had hemodialysis vascular access and were scheduled for treatment (AV-shunt and Hickman catheter) were excluded’’. Mention why!

Reply: Accordingly, the reason has been added to the Participants/Setting subsection of the METHODS (page 3). This is because catheter insertion was indicated in these cases due to the decision to undergo hemodialysis rather than delay treatment.

4. You should specify which approach did you follow in analysing data as there are different methods.

There are two general types of content analysis: conceptual analysis and relational analysis. Conceptual analysis determines the existence and frequency of concepts in a text. Relational analysis develops the conceptual analysis further by examining the relationships among concepts in a text. Each type of analysis may lead to different results, conclusions, interpretations, and meanings.

Reply: Accordingly, this description has been added to the “Data Collection and Analysis” subsection of the METHODS (pages 3 to 4). Accordingly, relevant details on the instructions and steps followed for conceptual analysis have been added to the manuscript.

5. Where? Again, this is a short cut information. The reader needs to have detailed information about setting and exactly how the participants were approached and interviewed. Who conducted the interview, were all the interviews conducted at the same time and day by the same researcher etc.. more details are needed please.

Reply: Accordingly, this description has been added to the “Data Collection and Analysis” subsection of the METHODS (pages 3 to 4). We have included details regarding the process of data collection by researchers.

6. Not sure what does that mean ... do you mean it was an individual interview to each patient please clarify need more details?

Reply: Accordingly, relevant descriptions have been added to the “Data Collection and Analysis” subsection of the METHODS (pages 3 to 4). We have added details regarding the process of data collection by the researchers and individual interview of each patient.

7. You mean with all participants or do you mean each individual interview. Also, you need to mention how was the interview initiated and continued give details please

Reply: This description has been added to the “Data Collection and Analysis subsection” of the METHODS (pages 3 to 4). We have added details regarding the process of data collection by the researchers and individual interview of each patient.

8. I would suggest using another expression such as deep investigation about perception or beliefs etc

Reply: This description has been revised in the Instrument subsection of the METHODS (Page 4). The indicated sentence has been revised to improve clarity.

9. Not sure what was mean this is not correctly written?? You might mean that the researcher developed the question which was used to lead the interview and to draw the data. If this was the case you need to mention in details how was the questions were formulated and from where did you draw the them was it from the literature or from similar r study etc … we never say that the researcher was the tool????

Reply: Accordingly, relevant descriptions have been added to the Instrument subsection of the METHODS (Page 4). The indicated sentence has been revised to improve clarity.

10. Method of data collection and analysis are missed and detailed describiton are needed to be added to this section. The method used for data analysis should be scientifecly described even if it was a qualtive study .. there are several methods to analyse the data to draw the themes of you study. It is a big fall of ther study if you did not follow one of them.

One example is Melis method of content analysis another one is Roger’s method so you should mention clearly which method did you follow and explain it in details

Reply: This description has been added to the “Data Collection and Analysis” subsection of the METHODS (pages 3 to 4). We have added relevant details regarding the instructions and steps for conceptual analysis.

11. Before mentioned the major themes there is a need to explain the word, phrases and then themes and subthemes found in the study this is a very short summary of your findings.
Reply: We have revised the Result section, adding more descriptions to explain their unique experience (pages 4 to 7).

12. General steps for conducting a conceptual content analysis:
1. Decide the level of analysis: word, word sense, phrase, sentence, themes.
2. Decide how many concepts to code for: develop pre-defined or interactive set of categories or concepts. Decide either: A. to allow flexibility to add categories through the coding process, or B. to stick with the pre-defined set of categories.
Reply: This description has been added to the “Data Collection and Analysis” subsection of the METHODS (pages 3 to 4). We have added relevant details regarding instructions and steps for conceptual analysis.

13. What was the subthemes found under this major findings?
Reply: Accordingly, we have revised the Result section, adding more descriptions to explain their unique experience (pages 4 to 7).

14. The same for this one see above notes

Reply: Accordingly, we have revised the Result section, adding more descriptions to explain their unique experience (pages 4 to 7).

15. The same comments see above please.
Reply: Accordingly, we have revised the Result section, adding more descriptions to explain their unique experience (pages 4 to 7).

16. Need further elaboration study findings and relating it with other research findings
Reply: Accordingly, relevant descriptions have been added to the DISCUSSION (pages 8 to 9). We have expanded the Discussion with regards to the study findings in relation to previous research findings.

17. Need to include how the findings would help to change the current practice at selected settings and what should be done further as an evidence-based practice to improve current condition. Also, you need to mention the limitations in your study. This would guide conducting of further research.
Reply: Accordingly, we have revised the DISCUSSION and CONCLUSION to reflect your clarifications (page 10). We have added relevant details on the study limitations and recommendations for future study.
