# Supplementary material for: The experience of urgent dialysis patients with end-stage renal disease: A qualitative study
Source: PLoS One. 2022 Jan 21;17(1):e0261941. doi: 10.1371/journal.pone.0261941 (PMC8782518; doi:10.1371/journal.pone.0261941)
Supplement: Attachment — Submitted filename: Response_to_Reviewers.docx [file pone.0261941.s003.docx]

Dear Editor:

We have modified the manuscript, which is now entitled “The experience of urgent dialysis patients with end-stage renal disease: a qualitative study” (PONE-D-21-04435), according to your comments. Our revisions are highlighted in red in the manuscript, and our modifications are detailed below. Thank you very much for reviewing our manuscript. We look forward to hearing from you.

Sincerely,

Kuei-Hui Chu

Reply to the reviewers’ comments:

1. It would be more precise to define age group of chosen patients.

Reply: Accordingly, relevant descriptions have been added to the INTRODUCTION and METHODS (page 2). The inclusion criterion was patients aged 60 years old or over.

2. You need to have a clear and precise inclusion criterion to inform the reader who were selected and considered eligible to be included. The information provided about sample is broad and not indicate any specification to the selected participants.

Reply: Accordingly, relevant descriptions have been added to the Participants/Setting subsection of the METHODS (page 2). We have added a clear and precise inclusion criterion and included details regarding age and disease characteristics.

3. “Patients who had hemodialysis vascular access and were scheduled for treatment (AV-shunt and Hickman catheter) were excluded’’. Mention why!

Reply: Accordingly, the reason has been added to the Participants/Setting subsection of the METHODS (page 3). This is because catheter insertion was indicated in these cases due to the decision to undergo hemodialysis rather than delay treatment.

4. You should specify which approach did you follow in analysing data as there are different methods.

There are two general types of content analysis: conceptual analysis and relational analysis. Conceptual analysis determines the existence and frequency of concepts in a text. Relational analysis develops the conceptual analysis further by examining the relationships among concepts in a text. Each type of analysis may lead to different results, conclusions, interpretations, and meanings.

Reply: Accordingly, this description has been added to the “Data Collection and Analysis” subsection of the METHODS (pages 3 to 4). Accordingly, relevant details on the instructions and steps followed for conceptual analysis have been added to the manuscript.

5. Where? Again, this is a short cut information. The reader needs to have detailed information about setting and exactly how the participants were approached and interviewed. Who conducted the interview, were all the interviews conducted at the same time and day by the same researcher etc.. more details are needed please.

Reply: Accordingly, this description has been added to the “Data Collection and Analysis” subsection of the METHODS (pages 3 to 4). We have included details regarding the process of data collection by researchers.

6. Not sure what does that mean ... do you mean it was an individual interview to each patient please clarify need more details?

Reply: Accordingly, relevant descriptions have been added to the “Data Collection and Analysis” subsection of the METHODS (pages 3 to 4). We have added details regarding the process of data collection by the researchers and individual interview of each patient.

7. You mean with all participants or do you mean each individual interview. Also, you need to mention how was the interview initiated and continued give details please

Reply: This description has been added to the “Data Collection and Analysis subsection” of the METHODS (pages 3 to 4). We have added details regarding the process of data collection by the researchers and individual interview of each patient.

8. I would suggest using another expression such as deep investigation about perception or beliefs etc

Reply: This description has been revised in the Instrument subsection of the METHODS (Page 4). The indicated sentence has been revised to improve clarity.

9. Not sure what was mean this is not correctly written?? You might mean that the researcher developed the question which was used to lead the interview and to draw the data. If this was the case you need to mention in details how was the questions were formulated and from where did you draw the them was it from the literature or from similar r study etc … we never say that the researcher was the tool????

Reply: Accordingly, relevant descriptions have been added to the Instrument subsection of the METHODS (Page 4). The indicated sentence has been revised to improve clarity.

10. Method of data collection and analysis are missed and detailed describiton are needed to be added to this section. The method used for data analysis should be scientifecly described even if it was a qualtive study .. there are several methods to analyse the data to draw the themes of you study. It is a big fall of ther study if you did not follow one of them.

One example is Melis method of content analysis another one is Roger’s method so you should mention clearly which method did you follow and explain it in details

Reply: This description has been added to the “Data Collection and Analysis” subsection of the METHODS (pages 3 to 4). We have added relevant details regarding the instructions and steps for conceptual analysis.

11. Before mentioned the major themes there is a need to explain the word, phrases and then themes and subthemes found in the study this is a very short summary of your findings.

Reply: We have revised the Result section, adding more descriptions to explain their unique experience (pages 4 to 7).

12. General steps for conducting a conceptual content analysis:

1. Decide the level of analysis: word, word sense, phrase, sentence, themes.

2. Decide how many concepts to code for: develop pre-defined or interactive set of categories or concepts. Decide either: A. to allow flexibility to add categories through the coding process, or B. to stick with the pre-defined set of categories.

Reply: This description has been added to the “Data Collection and Analysis” subsection of the METHODS (pages 3 to 4). We have added relevant details regarding instructions and steps for conceptual analysis.

13. What was the subthemes found under this major findings?

Reply: Accordingly, we have revised the Result section, adding more descriptions to explain their unique experience (pages 4 to 7).

14. The same for this one see above notes

Reply: Accordingly, we have revised the Result section, adding more descriptions to explain their unique experience (pages 4 to 7).

15. The same comments see above please.

Reply: Accordingly, we have revised the Result section, adding more descriptions to explain their unique experience (pages 4 to 7).

16. Need further elaboration study findings and relating it with other research findings

Reply: Accordingly, relevant descriptions have been added to the DISCUSSION (pages 8 to 9). We have expanded the Discussion with regards to the study findings in relation to previous research findings.

17. Need to include how the findings would help to change the current practice at selected settings and what should be done further as an evidence-based practice to improve current condition. Also, you need to mention the limitations in your study. This would guide conducting of further research.

Reply: Accordingly, we have revised the DISCUSSION and CONCLUSION to reflect your clarifications (page 10). We have added relevant details on the study limitations and recommendations for future study.
